# A small change in neuronal network topology can induce explosive synchronization transition and activity propagation in the entire network

**DOI:** 10.1038/s41598-017-00697-5

**Published:** 2017-04-03

**Authors:** Zhenhua Wang, Changhai Tian, Mukesh Dhamala, Zonghua Liu

**Affiliations:** 1 Department of Physics, East China Normal University, Shanghai, 200062 China; 2 Department of Physics and Astronomy, Neuroscience Institute, Center for Behavioral Neuroscience, Center for Nano-Optics, Center for Diagnostics and Theraputics, Georgia State University, Atlanta, Georgia 30032 USA

## Abstract

We here study explosive synchronization transitions and network activity propagation in networks of coupled neurons to provide a new understanding of the relationship between network topology and explosive dynamical transitions as in epileptic seizures and their propagations in the brain. We model local network motifs and configurations of coupled neurons and analyze the activity propagations between a group of active neurons to their inactive neuron neighbors in a variety of network configurations. We find that neuronal activity propagation is limited to local regions when network is highly clustered with modular structures as in the normal brain networks. When the network cluster structure is slightly changed, the activity propagates to the entire network, which is reminiscent of epileptic seizure propagation in the brain. Finally, we analyze intracranial electroencephalography (IEEG) recordings of a seizure episode from a epilepsy patient and uncover that explosive synchronization-like transition occurs around the clinically defined onset of seizure. These findings may provide a possible mechanism for the recurrence of epileptic seizures, which are known to be the results of aberrant neuronal network structure and/or function in the brain.

## Introduction

Explosive synchronization (ES) has received growing attention in network science since the discovery of its link with cascading failures of power grids^1–10^. This finding is important as it reveals a first-order synchronization transition in a network of phase oscillators where a second-order synchronization transition is predicted by the master stability function approach^11^. In their seminal work, Gomez and colleagues^1^ showed that ES occurs when (I) there is a positive correlation between the natural frequency of each oscillator and its degree and (II) the network is scale-free. Recently, a new framework for ES was suggested: ES occurs when the coupling strengths become proportional to the natural frequencies of coupled oscillators^4^. Furthermore, it was concluded that the condition for ES is in fact the *suppression rule* ^7^ and has been confirmed in a system with local adaptation control^8^. Despite these successes in associating abrupt dynamical behaviors to a mechanism, to our knowledge, there is not much work to address the abrupt transition to abnormal synchronization at the onset of epileptic seizures^12, 13^. This problem is not trivial as the abnormal synchronization may happen due to explosive spreading of an initial neuronal firing at the onset zone, whereas ES is due to an adiabatic increasing of coupling. Here, by modeling neurons and their networks, we aim to provide new insights into the interplay of the network structure and its network dynamics.

Recent work utilizing dynamical systems theory and network science approaches has increased our knowledge on brain functions and dysfunctions^14–16^. It is well accepted that synchronization of neurons play a major role in carrying out and sustaining the normal brain functions, such as perception, thoughts and motor behaviors^17^. For example, large-scale cortical synchronization^18, 19^ and synfire propagation occur in cognitive processes and the signal is carried by a wave of synchronous neuronal activity within a subset of network neurons^20–23^. Abnormal synchronization of neurons, on the other hand, is also the hallmarks of certain brain disorders, such as Parkinsons disease and epilepsy. The network science approaches have lead to uncovering the nature of brain network topology and its functions. The brain has been found to have a small-world network property, which is necessary for optimal communication of cortical neurons^24–28^. Another property is the modular structure with high clustering coefficient, which undertakes the functions in different brain areas such as the visual, olfactory and auditory senses etc^11, 14^.

Motivated by these findings on both ES and brain network topology, here we aim to provide useful insights into abrupt behaviors of neuronal synchronization as in epileptic seizures from normal states by studying how the interactions of brain neurons and network structure can facilitate explosive collective behaviors of coupled neurons. ES can be induced by an adiabatic increase of coupling strength. However, there is no evidence to show that a large synaptic strength change is required for the transition of the normal brain state to epileptic seizures^12^. In fact, after a brief period of recurrent epileptic seizures, the brain always returns to the normal functioning and spends most time in normal state. With this line of reasoning, we hypothesize that a small change in network topology is enough to set the transition to explosive synchronization of neurons and seizure-like propagation of neuronal activity. We examine action potential firing spreading over the various modeled networks under a slight change from critical network topology. Here, we also examine experimental data of epileptic seizures for explosive synchronization-like transitions and propagation.

## Results

### ES in neuronal networks

Overwhelming experimental evidence points to the idea that the brain and its functions can be best understood from a network perspective. The brain consists of a network of highly interconnected populations of neurons, coordinated functions of which in local small-scale regions and/or extended large-scale regions underlie our thoughts and actions. Brain networks can be derived from anatomical or physiological observations, resulting in structural or functional connectivity respectively. Structural connectivity (or network) describes anatomical connections (white matter projections) linking a set of neural elements. Functional connectivity is generally derived from time series observations and describes patterns of statistical dependence among neural elements. Many dynamical behaviors are the results of the interplay between the structure and function of the networks^11^. The brain has preferentially formed network clusters with modular network structure to support various brain functions^29, 30^. Here, we model networks of neurons to understand the interplay between the network cluster structure and normal functions and transitions to sudden or uncontrollable behaviors, like the epileptic seizures, and their propagation in the entire network. We describe below the details of key ingredients of our study: network topology and neuronal firing activity.

### Network topology

We first construct a random Erdös-Rényi (ER) network^31^ with size *N* = 1000 and average degree 〈*k*〉 = 8 and then gradually increase its clustering coefficient *C* by the Kim’s rewiring approach^32^ (see *Methods* for details). We find that a clear clustering will show up when *C* is increased to 0.5 and then the network will gradually become a modular topology with the further increase of *C* to *C* ≈ 0.75. Figures 2–6 in SM show the detailed network topologies for *C* = 0.5, 0.6, 0.7, 0.72 and 0.75, respectively, where the degree of modularity increases with *C*.

### Neuronal firing activity

We set an initial firing at an arbitrary node-*i* by choosing its initial condition as *u*(*i*) = 0.2 and *v*(*i*) = 0 and let other nodes be in excitable state by choosing their initial conditions as *u*(*j*) = *v*(*j*) = 0 with *j* ≠ *i*, i.e. only one initial firing node-*i*. Then, we let every node in the network be chosen as the initial firing node for one time. We pay attention to how this initial firing spreads to other parts of the network through the coupling links among the neurons.

**Figure 1:**
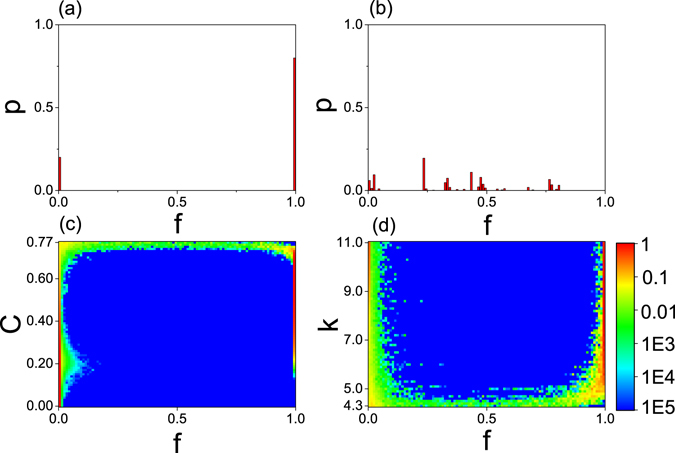
(**a**,**b**) *p* vs *f* where *f* represents the fraction of nodes ever being fired in one realization and *p* denotes the histogram of *f* in an interval of 0.01 when every node in the network has been chosen as the source node for one time. (**a**,**b**) Denote the cases of *C* = 0.5 and 0.75, respectively. (**c**,**d**) The distribution of *p* in the phase diagram of network states. (**c**) Denotes the dependence of *p* on the two parameters *C* and *f* for 〈*k*〉 = 8; (**d**) Denotes the dependence of *p* on the two parameters *k* and *f* for *C* = 0.7.

After building the models of networks of neurons, we now discuss neuronal firing propagation and mainly pay attention to how the network topology influences its dynamics through varying the clustering coefficient. For each realization of an initial firing, we let *f* represent the fraction of ever being fired nodes among the total *N* nodes. Thus, *f* will be different for different realizations. We let *p* denote the histogram of *f* in an interval of 0.01 for the *N* times realizations. Figure 1(a,b) show the results for *C* = 0.5 and 0.75, respectively. From Fig. 1(a) we see that there are only two parts with one with *f* ≤ 0.01 and another with *f* ≈ 1, indicating that the initial firing is either spread out globally or cannot spread out. From Fig. 1(b) we see that there are many different *f* but none of them with *f* ≈ 1, indicating that the firing spreadings are always limited in different local areas of the network for different realizations. Comparing Fig. 1(a) with (b), we see that only Fig. 1(b) is of the feature of normal brain network with diversity of patterns and no global synchronization, i.e. no abnormal synchronization.

To get more information on how the clustering coefficient *C* influences the firing spreading, Fig. 1(c) shows a phase diagram of the dependence of *p* on the parameters *f* and *C*. It is interesting to notice that the dynamical behaviors can be classified into three regions. In the first region with *C* < 0.13, *p* is nonzero only for a small *f*, indicating that the firing cannot be spread out in all the *N* realizations. In the second region with 0.13 < *C* < 0.73, *p* is nonzero only for the two ends of *f*, i.e. *f* being either close to zero or close to unity, indicating that the firing is similar to the case of Fig. 1(a). While in the third region with 0.73 < *C* < 0.77, *p* is nonzero for all the range of *f*, indicating that the firing is similar to the case of Fig. 1(b). In sum, we have the diversity of firing patterns only for a larger *C* but not for a middle or small *C*, implying that only the network with larger *C* can be used as a candidate to simulate the dynamics of brain with the modular structure.

**Figure 2:**
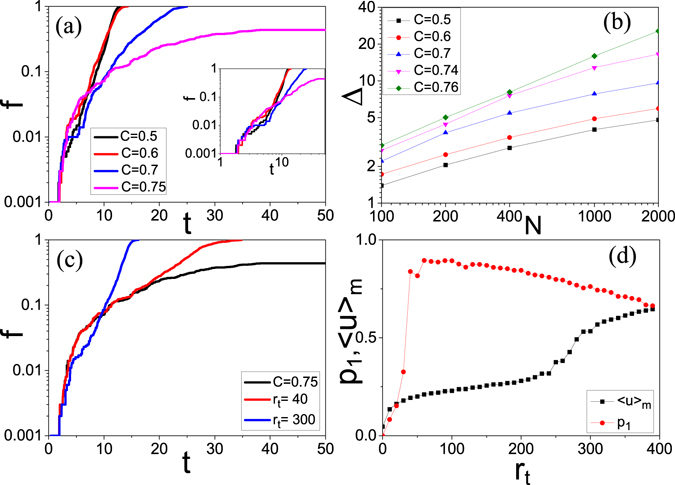
(**a**) Represents the typical cases for a chosen source node to spread its firing to the entire network where the four lines denote the situations of *C* = 0.5, 0.6, 0.7 and 0.75, respectively, and the inset is its log-log plot. (**b**) Δ versus *N* where the lines represent the cases of *C* = 0.5, 0.6, 0.7, 0.74 and 0.76, respectively. (**c**) The black line represents the case of *C* = 0.75 where the firing at chosen source node cannot be spread to the entire network. The red and blue lines represent the cases of rewiring the network with *C* = 0.75 for *r*_*t*_ = 40 and 300, respectively, where the firing at chosen source node can be now spread to the entire network and the rewired networks have *C* ≈ 0.72 and 0.5, respectively. (**d**) 〈*u*〉_*m*_ and *p*_1_ vs *r*_*t*_, where *p*_1_ represents the possibility for the firings at chosen source nodes to be spread to the entire network when every node in the network has been chosen as the source node for one time.

Except how large fraction of the network can be reached by an initial firing, another important question is how fast the spreading is. Figure 2(a) shows the evolution of the fraction *f* of active neurons in a linear-log plot where the four lines denote the cases of *C* = 0.5, 0.6, 0.7 and 0.75, respectively. It is easy to see that the three cases of *C* = 0.5, 0.6 and 0.7 can reach *f* = 1 but the case of *C* = 0.7 is much slower than the other two cases. To see it more clearly, we make a log-log plot in the inset. The two lines with *C* = 0.5 and 0.6 are approximately straight lines in the linear-log plot while the lines with *C* = 0.7 is an approximate straight line in the log-log plot, indicating that *f* increases with *t* in exponential for the cases of *C* = 0.5 and 0.6 and in a power-law for the case of *C* = 0.7. Generally, the exponential increase means a cascading spreading, which is closely related to ES^1, 7, 8^. While the power-law increase means that it may be fast increase in a local community and then slowly spread to other communities. For the case of *C* = 0.75, we see that *f* finally becomes stable and is smaller than 0.5, indicating that the firing spreading will be limited in local areas.

To provide a more rigorous definition of ES in Fig. 2(a), we recall that ES is in fact a process of explosive percolation (EP) in dynamical phase space^7^. An efficient approach to characterize the transition of EP is by two times^33–35^, the time *t*_0_ where the maximum cluster size equals $\sqrt{N}$ and the time *t*_1_ where the maximum cluster size equals *N*/2. Let Δ = *t*_1_ − *t*_0_. It is found that Δ is proportional to *N* ^*β*^, where *β* will be approximately unity for a second-order transition and smaller than unity for a first-order EP. That is, the value of *β* for the first-order transition of EP is much smaller than that of the second-order transition^33–35^. We here borrow this idea to characterize the firing spreading in Fig. 2(a). We keep *t*_0_ as the time for $f=\sqrt{N}/N$ and replace *t*_1_ as the time for *f* = 0.3*N*/*N*. Figure 2(b) shows the results for *C* = 0.5, 0.6, 0.7, 0.74 and 0.76, respectively. We see that the slopes of the lines with *C* = 0.74 and 0.76 are much larger than that of the lines with *C* = 0.5, 0.6 and 0.7, confirming that their corresponding transitions are fundamentally different, i.e. the first-order and second-order transitions, respectively.

We now confirm that the network of *C* = 0.75 has the features of normal brain network. Then, an interesting question is how can we make this network show the firing ES? To figure out the answer, we randomly rewire a small fraction of its links without the condition of increasing network’s clustering coefficient, in contrast to the Kim’s rewiring approach^32^. We find that this randomly rewiring does not influence its modular structure very much, but results in more links between the communities and thus make the initial firing easily spread to the entire network. The red and blue lines in Fig. 2(c) show the results for the rewiring times *r*_*t*_ = 40 and 300, respectively. It is easy to see that both of them can reach *f* = 1, i.e. global spreading. From Fig. 2(c) we also notice that only the line with rewiring 300 times is an approximate straight line, i.e. exponential, indicating its equivalence to the cases of *C* = 0.5 and 0.6 in Fig. 2(a). Figures 7 and 8 in SM show the network topologies of *r*_*t*_ = 40 and 300, respectively. We have to point out that it is also possible to have *f* < 1 for the cases of *r*_*t*_ = 40 and 300 in other realizations. To see the relationship between the global spreading and rewiring times *r*_*t*_ more clear, we let *p*_1_ represent the possibility of *f* = 1 when every node in the network has been chosen as the initial firing for one time. The “circles” in Fig. 2(d) shows the dependence of *p*_1_ on *r*_*t*_. It is easy to see that *p*_1_ has a big jumping at a critical point ${r}_{t}^{c}\approx 25$ where the clustering coefficient is slightly reduced to *C* ≈ 0.72, indicating that a slight change of network results in a big change in firing propagation.

**Figure 3:**
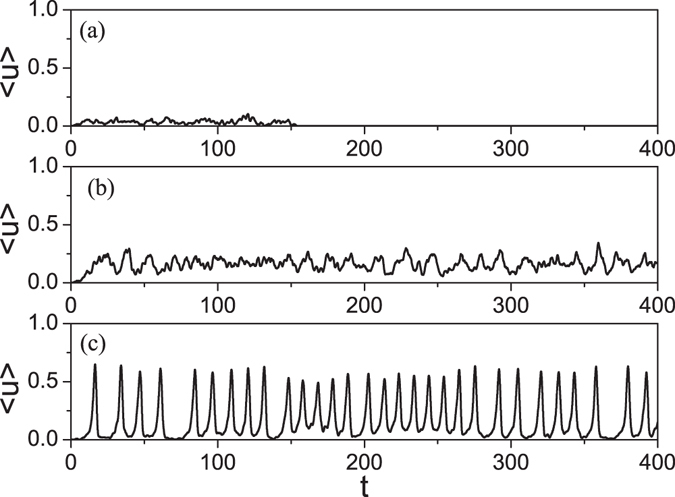
(**a**) How a stimulating firing at the network of *C* = 0.75 spreads to other nodes where 〈*u*〉 is the average on all the *N* nodes. (**b**,**c**) Represent the cases of rewiring the network with *C* = 0.75 for *r*_*t*_ = 40 and 300, respectively, where the rewired network has *C* ≈ 0.72 and 0.5, respectively.

Furthermore, we have calculated the evolution of the average $\langle u\rangle =\frac{1}{N}{\sum }_{i=1}^{N}u(i)$. The active (firing) nodes at time *t* will not be a large fraction of the total nodes if 〈*u*〉 is a small quantity, but a large fraction if 〈*u*〉 is large. Especially, there will be a strong firing synchronization if 〈*u*〉 has a regular oscillatory behavior, like the spikes of a single neuron. Figure 3(a–c) represent the evolution of 〈*u*〉 for the cases of *r*_*t*_ = 0, 40 and 300, respectively, from the network with *C* = 0.75. From Fig. 3(a) we see that 〈*u*〉 becomes zero after *t* = 150, indicating that the firings are restricted in local areas and finally dead, see Fig. 9(b) in SM for details. From Fig. 3(b) we see that the oscillation death in Fig. 3(a) is replaced by a self-sustained oscillation. Especially, we observe a strong or abnormal synchronization in Fig. 3(c) where most of the neurons have in step firing and refractory, see Fig. 9(d) in SM for details. We should point that the neurons cannot be all synchronized at the same time as that will make them go to the the refractory at the same time and then result in death.

We notice from Fig. 3(b,c) that the average 〈*u*〉 is an oscillation with multiple peaks and the heights of peaks are related to the firing synchronization of the network. Thus, we introduce 〈*u*〉_*m*_ to measure the degree of firing synchronization, with 〈*u*〉_*m*_ being the average of the heights of peaks. A large 〈*u*〉_*m*_ represents the case of strong firing synchronization and global firing spreading. The “squares” in Fig. 2(d) shows the dependence of 〈*u*〉_*m*_ on *r*_*t*_. We see that 〈*u*〉_*m*_ can be separated into two parts, i.e. smaller 〈*u*〉_*m*_ for *r*_*t*_ < 250 and larger 〈*u*〉_*m*_ for *r*_*t*_ > 290, indicating that the case with *r*_*t*_ > 290 is of both exponential fast spreading (see Fig. 2(c)) and abnormal synchronization (see Fig. 3(c)).

It is maybe also interesting to investigate how the average degree influences the firing spreading, as this type of networks can undergo phase transitions in their dynamics at some critical average connectivity^36^. For this purpose, we have fixed the clustering coefficient at *C* = 0.7 (smaller than the critical *C* ≈ 0.73 for 〈*k*〉 = 8 in Fig. 1(c)) and then change the average degree. We find that there is a critical 〈*k*_*c*_〉 ≈ 5.0 to separate the first-order and second-order propagation. Figure 1(d) shows the result where there is a diversity of firing patterns for *k* < 〈*k*_*c*_〉 and only two or a few patterns for *k* > 〈*k*_*c*_〉. Comparing Fig. 1(d) with Fig. 1(c), we see that they are similar, indicating that the average degree has the similar effect as the clustering coefficient.

### Explosive synchronization-like transitions to epileptic seizures in an experimental data

**Figure 4:**
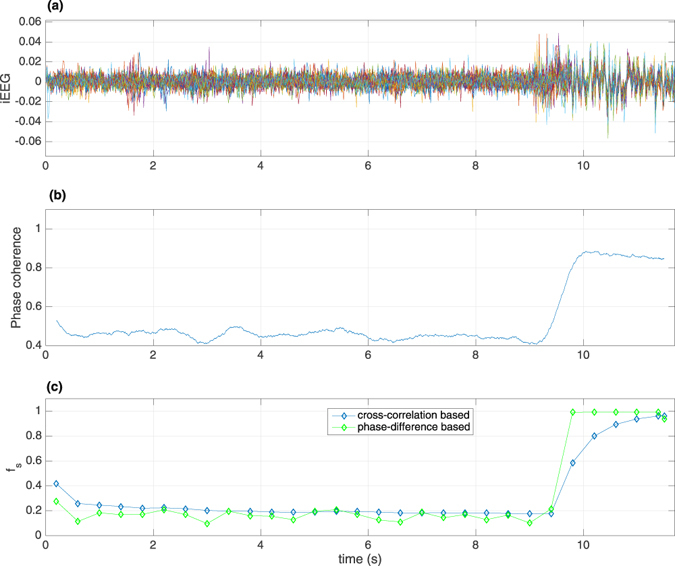
Shows (**a**) 118 channel time series of IEEG recordings from an epilepsy patient with an epileptic seizure event occurring at around 9 seconds, (**b**) Kuramoto phase coherence over time, and (**c**) cross-correlation based (blue) and phase-difference-based (green) *f*_*s*_(*t*) versus time *t* by setting the threshold *R*_0_ = 0.4, where *f*_*s*_(*t*) represents the fraction of *R*_*ij*_ ≥ *R*_0_ in the possible *N* × *N* pairs.

To connect the above theoretical results of explosive synchronization-transitions, we re-analyzed previously reported de-identified IEEG data during an epileptic seizure^13^ and looked at the nature of the seizure onset (Fig. 4). Figure 4(a) shows multichannel IEEG recordings with a seizure event clinically determined to be at around 9.0 seconds. The IEEG recordings were done with combinations of depth and subdural electrodes at a sampling rate of 500 Hz from a patient undergoing clinical evaluation for epilepsy surgery at Emory University Hospital in Atlanta. This study was approved by the local Institutional Review Board. Typically, most of the patients with medication resistant epilepsy undergo IEEG recordings and surgery for the treatment.

In Fig. 4(a), we show the 118 electrode recordings of IEEG time series with an epileptic seizure starting at around 9 *sec*. It is easy to see that the amplitudes of time series are small when *t* < 9 and then they rapidly increase to larger values between 9 < *t* < 9.5, marking the onset of seizure. This kind of rapidly increasing closely resembles with the process of explosive synchronization (ES)^1, 7, 8^. To assess the nature of the seizure onset, here, we compute the overall phase coherence across all electrode recordings (Fig. 4(b)) and cross-correlation based and pairwise phase-difference (*R*_*ij*_) based measures of fractions *f*_*s*_(*t*) of recording pairs that are significantly above the baseline level or threshold *R*_0_ = 0.4 (Fig. 4(c)). See *Methods* for details. We find that most elements of the matrix *R*_*ij*_ are of small values before the onset of seizure and then they quickly increase to close to unity at the onset of seizure, indicating the appearance of abnormal synchronization. Figure 10 in SI show the results at different times. To measure how fast the abnormal synchronization is developed from the local synchronization, we set a threshold *R*_0_ = 0.4 and calculate how many pairs of *R*_*ij*_ are larger than *R*_0_ at time *t*. Let *f*_*s*_(*t*) represent the fraction of *R*_*ij*_ ≥ *R*_0_ in the possible *N*(*N* − 1) pairs, see *Methods* for details. Figure 4(b,c) shows the evolution of phase coherence and *f*_*s*_(*t*) with *t*. It is easy to see that *f*_*s*_(*t*) has an abrupt behavior around the onset of epileptic seizure with *t* ≈ 9.5, mimicking the main feature of ES. The abrupt transitions in Fig. 4(b,c) are similar to Fig. 2(d).

### Network topology and neuronal activity spreading between clusters of interacting neurons

**Figure 5:**
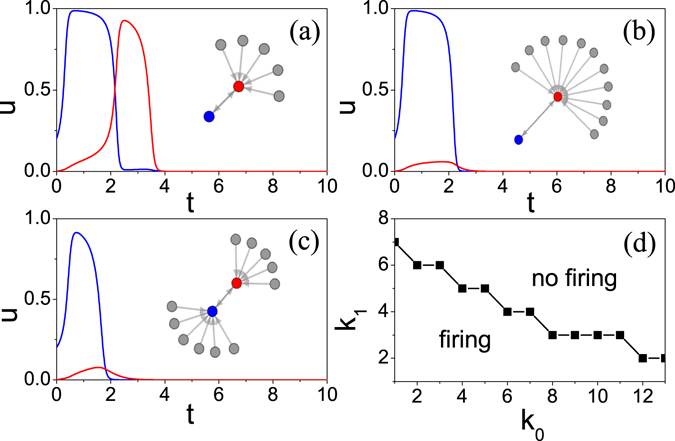
(**a**–**c**) How a stimulating firing (blue) induces a firing (red) at the acceptor for different configurations where the inset represents the configuration of the two connected neurons with the “blue” node being the source and the “red” node being the acceptor. The source and acceptor nodes have their degrees *k*_0_ = 1 and *k*_1_ = 6 in (**a**), respectively, *k*_0_ = 1 and *k*_1_ = 12 in (**b**), respectively, and *k*_0_ = 7 and *k*_1_ = 6 in (**c**), respectively. (**d**) The line with “squares” represents the boundary where the firing at the “red” node can be induced below the boundary but not above the boundary.

To understand the mechanism of the influence of network topology revealed in Figs 1, 2 and 3, first, we have to understand how a firing activity spreads to its neighboring nodes. For this purpose, we consider different connection patterns and then figure out the rules for successful spreading. The insets of Fig. 5(a–c) show three typical patterns of connection where the “blue” node represents the source node with a firing from the initial condition of *u*(0) = 0.2 and *v*(0) = 0 and the “red” node denotes the target or acceptor with the initial condition of *u*(0) = *v*(0) = 0. We want to see if there is a successful transmission of firing activity from the source neuron to the acceptor neuron. Our numerical simulations show that a firing activity is induced at the acceptor in Fig. 5(a) but not in Fig. 5(b,c), where the blue and red curves represent the evolution of *u*(*t*) at the source and acceptor nodes, respectively. Comparing Fig. 5(a) with (b) we see that their source nodes have the same degree *k*_0_ = 1 but their acceptors have different degrees, i.e. *k*_1_ = 6 in (a) and *k*_1_ = 12 in (b), indicating that a smaller *k*_1_ supports the firing activity spreading while a larger *k*_1_ does not. While comparing Fig. 5(a) with (c), we see that their acceptors have the same degree *k*_1_ = 6 but their source nodes have different degrees, i.e. *k*_0_ = 1 in (a) and *k*_0_ = 7 in (c), indicating that a smaller *k*_0_ supports the firing spreading activity while a larger *k*_0_ does not. Thus, both *k*_0_ and *k*_1_ are the key elements to the firing spreading activity. Further, we also notice that *k*_0_ and *k*_1_ have different functions in preventing the firing spreading activity. A larger *k*_0_ reduces the amplitude of the initial firing activity (see the blue curve in Fig. 5(c)) and thus makes the firing spreading to be more difficult, while a larger *k*_1_ makes the acceptor neurons difficult to firing activity.

Moreover, Fig. 5(c) tells us an important information that its initial firing is different from that in Fig. 5(a,b), indicating that the initial firing can be also influenced by its neighbors. This difference asks us a key question: what are the right initial conditions for an arbitrary node in the network to be induced for action potential firing? Its answer is related not only to the initial firing but also to the spreading of activity. To figure out this answer, we turn to the neuron’s dynamics Eq. (2). To stimulate action potential firing of a node-*i*, we choose initial conditions of *u*(*i*) ≥ *u*_0_ and *v*(*i*) ≥ *v*_0_ so that *du*_*i*_/*dt* > 0 and *dv*_*i*_/*dt* > 0 in Eq. (2). For simplicity, we set *v*(*i*) = *v*_0_ = 0 as an example. For a single neuron, *du*_*i*_/*dt* > 0 of Eq. (2) gives *u*_0_ ≥ *b*/*a* ≈ 0.083. For a node-*i* in the network with *k*_*i*_, *du*_*i*_/*dt* > 0 of Eq. (2) gives $({u}_{i}-1)({u}_{i}-b/a)+c\varepsilon {k}_{i} < 0$. Taking *k*_*i*_ = 〈*k*〉 = 8 as an example, we obtain *u*_0_ ≈ 0.147. In our numerical simulations, we use the initial conditions *u*(*i*) = 0.2 > *u*_0_ and *v*(*i*) = 0 for the source node so that an initial firing can be successfully generated.

**Figure 6:**
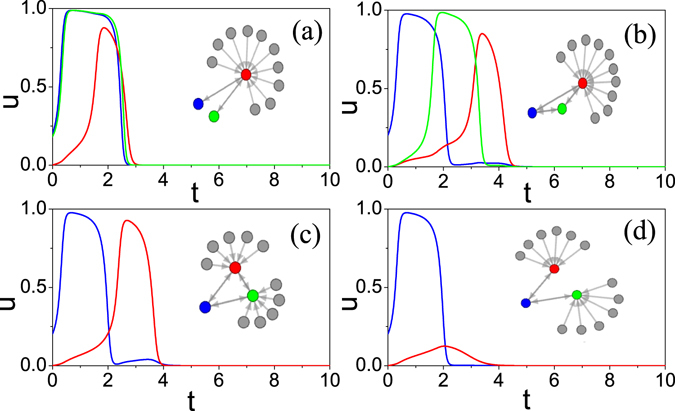
(**a**) Two source nodes (the “blue” and “green” nodes) drive a common acceptor (the “red” node) where the acceptor has degree *k*_1_ = 12. The blue and green lines represent the two stimulating firings while the red line denotes the induced firing. To distinguish the two stimulating firings, we let their initial conditions as *u*(0) = 0.2, *v*(0) = 0 (for the “blue” node) and *u*(0) = 0.18, *v*(0) = 0 (for the “green” node), respectively. (**b**) A source node (the “blue” node) drives a “green” node first and then they work together to drive a common acceptor (the “red” node) where the acceptor has degree *k*_1_ = 12. (**c**,**d**) A source node (the “blue” node) drives two acceptors (the “red” and “green” nodes) where each acceptor has degree *k*_1_ = 7. The two acceptors are connected in (**c**) but separated in (**d**).

Figure 5(d) shows the dependence of firing spreading on *k*_0_ and *k*_1_ where the line with “squares” is the boundary, i.e. a firing at the acceptor can be induced in the region below the boundary but not in the region above the boundary. A slight complicated connection patterns are the cases where two source nodes connect one acceptor or one source node connects two acceptors. For the former, Fig. 6(a) shows a typical example where both the “blue” and “green” nodes are the source nodes with firings and they work together to drive the “red” acceptor to generate action potential firing. We notice that the acceptor’s degree *k*_1_ = 12 is much larger than the boundary *k*_1_ < 7 in Fig. 5(d), implying that two source nodes can extend boundary *k*_1_ to a larger value. Figure 6(b) shows a different case where there is only one source node (the “blue” one), but it induces a firing at the “green” node first and then they work together to induce a firing at the “red” node. We notice from Fig. 6(b) that there is an overlap between the blue and green firing curves, which is equivalent to the function of two source nodes in Fig. 6(a) and thus can induce a firing at the “red” node. For the latter, Fig. 6(c,d) show two examples where each acceptor has the degree *k*_1_ = 7 and there is a connection between the two acceptors in Fig. 6(c) but no connection in Fig. 6(d). Notice that *k*_1_ = 7 is the boundary degree for *k*_0_ = 1 in Fig. 5(d). Thus, the connection between the two acceptors in Fig. 6(c) influences the boundary condition, indicating that the connection has produced a positive effect on the firing spreading.

Based on these results, we can now explain the observations in Fig. 1(c). When *C* < 0.13, the network is random homogeneous with no modularity. Both its *k*_0_ and *k*_1_ are around 8, which is located in the region of no firing in Fig. 5(d) and thus the initial firing cannot spread out. When 0.13 < *C* < 0.73, the network becomes random and homogeneous with modular structure. In each module or community, the initial firing node is likely to display similar activity as in Fig. 6(b,c), which results in the global firing spreading. When 0.73 < *C* < 0.77, the network is highly modular with only one or a few connections between different communities, see Fig. 6 in SM. In this sense, for a neuron network, its firing spreading in a community is similar to the cases of 0.13 < *C* < 0.73 while the firing spreading between communities is similar to the cases of *C* < 0.13, which results in the diversity patterns but with no global spreading in the cases of 0.73 < *C* < 0.77. In sum, these results tell us that in a real neuronal network, the induced firing is not only related to the initial firing node but also related to the activity from the surrounding neurons.

## Discussion

Here, we use a simple model of networks of neurons (2) and show that the abrupt dynamical behaviors of the networks and network activity resemble what really happens in real brain during a course of an epileptic seizure event. The normal brain network is highly clustered with modular structure and has small world topology for optimal communication as many recent studies suggest. Here, we show that these features in (2) occur when *C* is around 0.75, see Fig. 6 in SI. Figures 1 and 2 also tell us that the highly clustered topology is necessary for the stability of local sustained activities. Another key feature of brain network is its self-organization criticality (SOC)^37, 38^, implying that the brain network works at its critical point. This feature can be confirmed in Fig. 2(d) by the narrow range of ${r}_{t} < {r}_{t}^{c}$, i.e. before the jumping of *p*_1_. Once ${r}_{t} > {r}_{t}^{c}$, firing propagation will be substantially different, indicating that a small change of network results in a big change of system’s behavior, i.e. the marking of SOC.

A key result from (2) is that firing ES can be induced by slightly rewiring the network links, which can be considered as an alternative way to the coupling induced ES^1, 7, 8^. There is a common point between firing ES and the coupling induced ES. For the former, high clustering guarantees firing ES; while for the latter, the suppression rule prevents the appearance of ES^7^, i.e. clustering corresponding to suppression rule. This finding also tells us that the brain network is more complicated than what is generally thought of. Considering that communication between two neighboring neurons is implemented through their firing activities and interactions, the spreading of synchronization can be thought of equivalent to the spreading of firings, i.e. the spreading of an initial firing from a focal region to the entire network. In this sense, we may assume that the appearance of firing ES in our neural network corresponds to the onset of epileptic seizure in brain network.

We have to point that the *x* axis in Fig. 4(a–c) is neither coupling strength nor rewiring times, but time. The coupling strengths (i.e. synaptic strengths) between neurons are not directly accessible, therefore there is no direct link experimentally established between coupling strength and epileptic seizure events. Traditionally, a big change in dynamics needs a big external force. Using this idea to brain, the onset of epileptic seizure will need a big interaction, which will result in unrecoverable damages and thus cannot be recovered in a short time. This is definitely not true as patients generally return to a normal state after a brief period of a seizure. This notion is consistent with what we found from our simulation: the firing ES can be induced by a small change in network structure and thus the system can get back to the previous normal state.

The next key question is: how an acceptor neuron-*j* with *u*(*j*) = *v*(*j*) = 0 can be induced to firing an action potential? To figure out the answer, we consider a specific case with a condition with one of its neighbors, node-*i*′, is in firing mode. The condition *du*_*j*_/*dt* > 0 in Eq. (2) gives

1 $${u}_{j}(1-{u}_{j})({u}_{j}-b/a)+c\varepsilon [({u}_{{i}^{^{\prime} }}-{u}_{j})+\sum _{i\ne i^{\prime} }{A}_{ij}({u}_{i}-{u}_{j})] > 0$$

When *u*_*j*_ is small and *k*_*i*_ = 1, the first term of Eq. (1) can be approximately taken as *u*_*j*_ (*u*_*j*_ − *b*/*a*) and the second term of Eq. (1) can be approximately taken as $c\varepsilon {u}_{{i}^{^{\prime} }}\approx c\varepsilon$. Eq. (1) becomes ${u}_{j}^{2}-b{u}_{j}/a+c\varepsilon  > 0$, which is always true as ${(b/a)}^{2}-4c\varepsilon  < 0$ for the chosen parameters in Eq. (2). Thus, *u*_*j*_ will increase with time. After *u*_*j*_ > *b*/*a*, a firing will be induced. While for the case of *k*_*i*_ > 1, the term ${\sum }_{i\ne {i}^{^{\prime} }}{A}_{ij}({u}_{i}-{u}_{j})$ will be negative when *u*_*j*_ becomes positive, which make the second term of Eq. (1) be less than *cε* such as becoming *δcε* with *δ* < 1. In this sense, for a larger *k*_*i*_, it is possible for (*b*/*a*)^2^ − 4*δcε* = 0 and then *u*_*j*_ will stop to increase, implying no firing will be induced. More complicated cases can be similarly analyzed. In sum, active neighbors take a positive role in firing spreading while the inactive neighbors take a negative role. Therefore, a successful spreading from node-*i* to node-*j* depends on both the number of firing neighbors and the number of inactive neighbors.

In conclusion, we have presented a model of neuronal networks to study abrupt seizure-like propagation induced as a result of the interplay of network topology and neuronal activity in the neighborhood. Through this model, we show that firing spreading is limited in one or a few local regions when the network is highly clustered with modular structures as in the normal brain function. However, this local behavior is fragile and can easily induce a firing ES in a slightly rewired network. This behavior is similar to the onset of epileptic seizure which can originate in a focally local region and then spreads to other regions of brain network. A correspondence between the firing of ES and the onset of seizure is shown by using real experimental data. These findings provide useful insights into the mechanism for the recurrence of epileptic seizures.

## Methods

### Neuron model

Let each node on the constructed network represent an excitable neuron and the coupling be bidirectional. In this way, each neuron-*i* will be connected to its *k*_*i*_ neighbors. In detail, we let each node be a modified version of a piecewise linearized FitzHugh-Nagumo model as follows^39, 40^

2 $$\begin{array}{rcl}\frac{d{u}_{i}}{dt} & = & -\frac{1}{\varepsilon }{u}_{i}({u}_{i}-\mathrm{1)}({u}_{i}-\frac{{v}_{i}+b}{a})+c\sum _{j=1}^{N}{A}_{ij}({u}_{j}-{u}_{i})\\ \frac{d{v}_{i}}{dt} & = & f({u}_{i})-{v}_{i}\end{array}$$

where *A*_*ij*_ is the conjunction matrix with *A*_*ij*_ = 1 if two neurons *i* and *j* are connected, and *A*_*ij*_ = 0 otherwise. *f*(*u*) is chosen as the following function: *f*(*u*_*i*_) = 0 for *u*_*i*_ < 1/3, $f({u}_{i})=1-6.75{u}_{i}{({u}_{i}-\mathrm{1)}}^{2}$ for 1/3 ≤ *u*_*i*_ ≤ 1, and *f*(*u*_*i*_) = 1 for *u*_*i*_ > 1. *u*_*i*_ and *v*_*i*_ represent the fast and slow variables, respectively. *ε* is a small parameter which warrants a clear separation between the slow and fast time scales. The system parameters are kept throughout this paper as *ε* = 0.04, *a* = 0.84, *b* = 0.07, and *c* = 0.17 just for the sake that the local cell follows excitable dynamics.

### Experimental IEEG data

De-identified IEEG data from one epilepsy patient, previously published in ref. 13 was re-analyzed in this current study. The data were recorded at a sampling rate of 500 Hz by using combination of depth, subdural grid and strip electrodes, a total of 118 electrodes. In this segment, the clinically defined onset of seizure occurred at around 9 sec (Fig. 4(a)). For further information about the data and patients, see refs 13, 41.

To measure the phase correlation between time series *s*_*i*_ and *s*_*j*_, we first extract their phases *θ*_*i*_(*t*) and *θ*_*j*_(*t*) by using the approach in ref. 42. We compute three quantities: Kuramoto’s phase coherence (Fig. 4(b)), fraction of phase-synchronized or cross-correlated oscillator pairs (Fig. 4(b,c)). The phase-difference base local order parameter

3 $${R}_{ij}=|\mathop{\mathrm{lim}}\limits_{T\to \infty }\frac{1}{T}{\int }_{t}^{t+T}{e}^{i[{\theta }_{i}(t)-{\theta }_{j}(t)]}dt|,$$

where *T* is the time window to measure the correlation. *R*_*ij*_ will be in between 0 and 1 and a larger value of *R*_*ij*_ represents a stronger phase synchronization.

To measure how fast the abnormal synchronization is developed from the local synchronization, we set a threshold *R*_0_ = 0.4 and calculate how many pairs of *R*_*ij*_ are larger than *R*_0_ at time *t*. Letting *f*_*s*_(*t*) represent the fraction of *R*_*ij*_ ≥ *R*_0_ in the possible *N*(*N* − 1) pairs, we have

4 $${f}_{s}(t)=\frac{1}{N(N-\mathrm{1)}}\sum _{i=1}^{N}\sum _{j\ne i}H({R}_{ij}-{R}_{0})$$

where *H*(*x*) is the step function with *H*(*x*) = 1 if *x* ≥ 0 and *H*(*x*) = 0 otherwise. Figure 4(c) is calculated by Eq. (4).

### Clustering coefficient

The network is constructed as follows. We first construct a random ER network^31^ with size *N* = 1000 and average degree 〈*k*〉 = 8. Its clustering coefficient can be calculated as follows^31^

5 $$C=\frac{1}{N}\sum _{i=1}^{N}\frac{{E}_{i}}{{k}_{i}({k}_{i}-1)/2}$$

where *k*_*i*_ is the degree of node-*i* and *E*_*i*_ is the number of edges among the neighbors of node-*i*.

### Kim’s rewiring approach

Then, we change its clustering coefficient by the Kim’s rewiring approach^32^, which has the advantage that the degree of each node will remain unchanged when we change its clustering coefficient. The algorithm of the rewiring approach can be stated as follows: randomly choose two links, one connecting nodes A and B, and the other C and D. Each node changes its partner and the original links A–B and C–D are altered to A–D and B–C. The link exchange trial is accepted only when the new network configuration goes to a higher clustering coefficient.

## Electronic supplementary material

Supplementary info
