# Supplementary material for: A small change in neuronal network topology can induce explosive synchronization transition and activity propagation in the entire network
Source: Sci Rep. 2017 Apr 3;7:561. doi: 10.1038/s41598-017-00697-5 (PMC5428839; doi:10.1038/s41598-017-00697-5)
Supplement: Supplementary file 1 — Supplementary info [file 41598_2017_697_MOESM1_ESM.pdf]

A small change in neuronal network topology can induce explosive synchronization transition and activity propagation in the entire network

Zhenhua Wang[1], Changhai Tian[1], Mukesh Dhamala[2] and Zonghua Liu[1*]

I. NETWORK TOPOLOGIES

Fig. 1 shows a typical ER network with size $N = 1000$ and average degree $\langle k \rangle = 8$, where its clustering coefficient is $C \approx 0.007$.

Figs. 2-6 show the typical networks rewired from the ER network of Fig. 1 by the Kim's rewiring approach [1], where the clustering coefficients of Figs. 2-6 are $C \approx 0.5, 0.6, 0.7, 0.72$ and 0.75, respectively.

Figs. 7-8 show two typical networks rewired from the network of Fig. 6 with $C \approx 0.75$ by the random rewiring approach for $r_t = 40$ and 300, respectively, where their clustering coefficients are $C \approx 0.72$ and 0.5, respectively. The common point between the Kim's rewiring approach and the random rewiring approach is that they both do not change the degree distribution. Their main difference is that the former only chooses those rewiring to increase the clustering coefficient and leave it unchange otherwise, while the later uses all the randomly chosen rewiring no matter it will increase or decrease the clustering coefficient.

II. SPREADING OF AN INITIAL FIRING IN REWIRED NETWORK FROM THAT ONE WITH $C = 0.75$

Fig. 1(d) in main text shows that an initial firing cannot spread to the entire network when $C = 0.75$, indicating that the firing spreading will be limited in one or a few local modules. To show the firing spreading more clear, we simplify the dynamical process by letting $s(i) = 1$ when $u(i) > u_0$ and $s(i) = 0$ otherwise, where $u_0 = 0.5$ is the firing threshold. We randomly choose a node i_0 to have an initial firing from the initial condition $u(i_0) = 0.2$ and $v(i_0) = 0$ and let other nodes be excitable with the initial condition $u(i_0) = 0$ and $v(i_0) = 0$. Fig. 9(a) shows the dynamical process of $s(i)$ from the initial firing, where a point is put in the panel when $s(i) = 1$ and leave it empty when $s(i) = 0$. We see that all the points disappear when $t > 150$, implying the firing death.

Fig. 9(b) shows the reordering of Fig. 9(a) by the total number of firings at each node in the evolution period, with descending order. It is clear that the initial firing is located in the nodes around $i = 500$ and limited there for a finite time. After that, the firings are spread to the nodes with $i < 300$ and then dead at $t \approx 150$. A characteristic feature is that the firings are never spread to the nodes with $i > 750$, indicating that the firing spreading is non-global with $f < 1$.

Then, we let the network with $C = 0.75$ be randomly rewired for $r_t = 40$ and 300, respectively, and do the same dynamical process as in Fig. 9(a). Fig. 9(c) and (d) represent the cases of randomly rewiring the network with $C = 0.75$ for $r_t = 40$ and 300, respectively, where the rewired network has $C \approx 0.72$ and 0.5, respectively. It is easy to see that the rewiring has made the firing spreading be global. Comparing Fig. 9(c) with (d) we see that the firings are weak synchronized in Fig. 9(c) but strong synchronized in Fig. 9(d), indicating that the rewired topology in Fig. 9(d) may represent the brain network of epileptic seizure.

III. PHASE CORRELATION MATRIX OF EXPERIMENTAL DATA

Figure 10 shows the evolution of phase correlation matrix from experimental data where the time interval is taken as $0.4s$. The elements of R_{ij} is defined in the *Methods* of main text.

References

[1] Kim B. J. Performance of networks of artificial neurons: The role of clustering. *Phys. Rev. E* **69**, 045101(R) (2004).

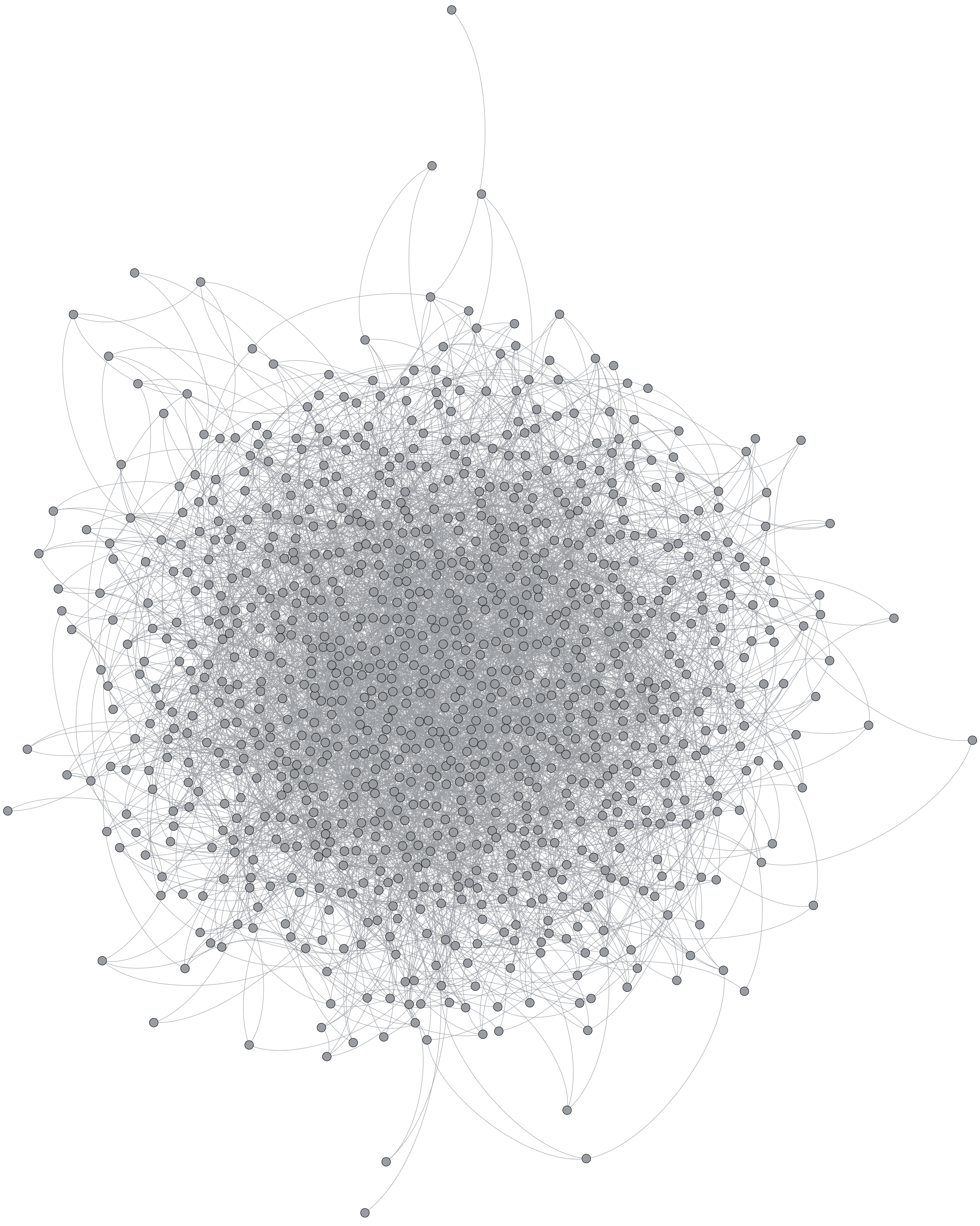

FIG. 1: (Color online.) **Topological structure of the original ER network with clustering coefficient** $C \approx 0.007$. The network has size $N = 1000$ and average degree $\langle k \rangle = 8$.

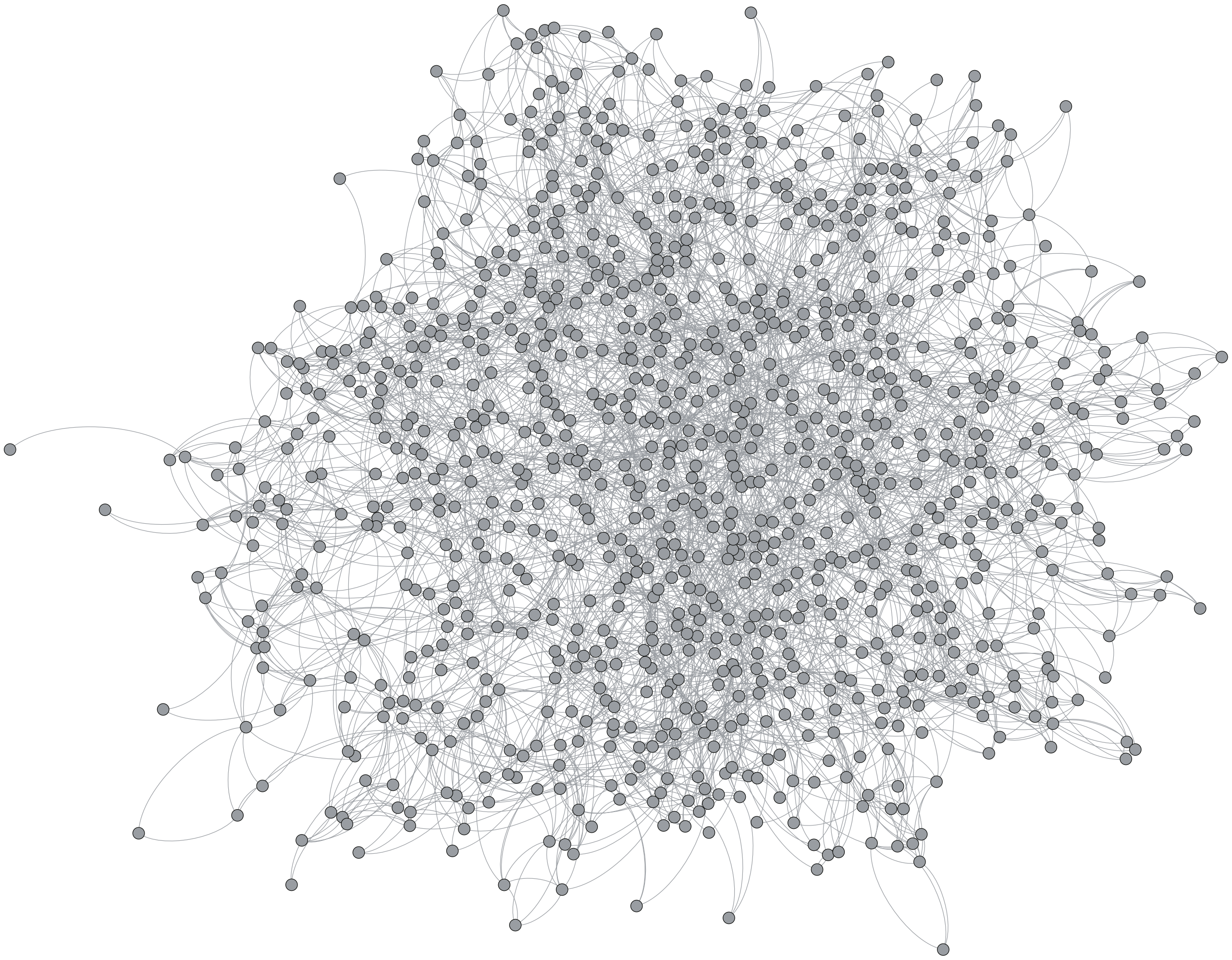

FIG. 2: (Color online.) **Topological structure of the network rewired from the ER network of Fig. 1 by the Kim's rewiring approach**. The rewired network has the clustering coefficient $C \approx 0.5$.

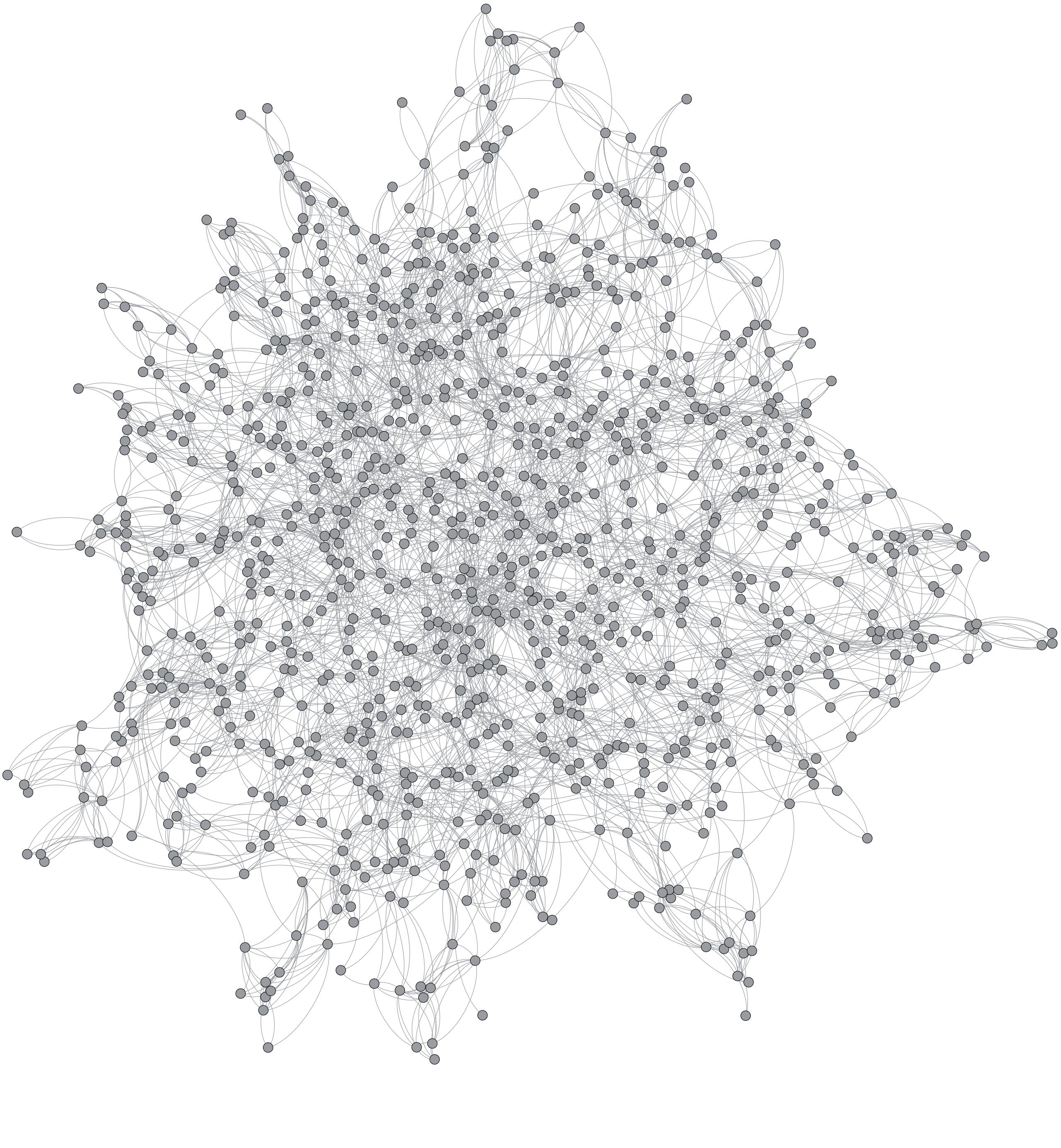

FIG. 3: (Color online.) **Topological structure of the network rewired from the ER network of Fig. 1 by the Kim's rewiring approach**. The rewired network has the clustering coefficient $C \approx 0.6$.

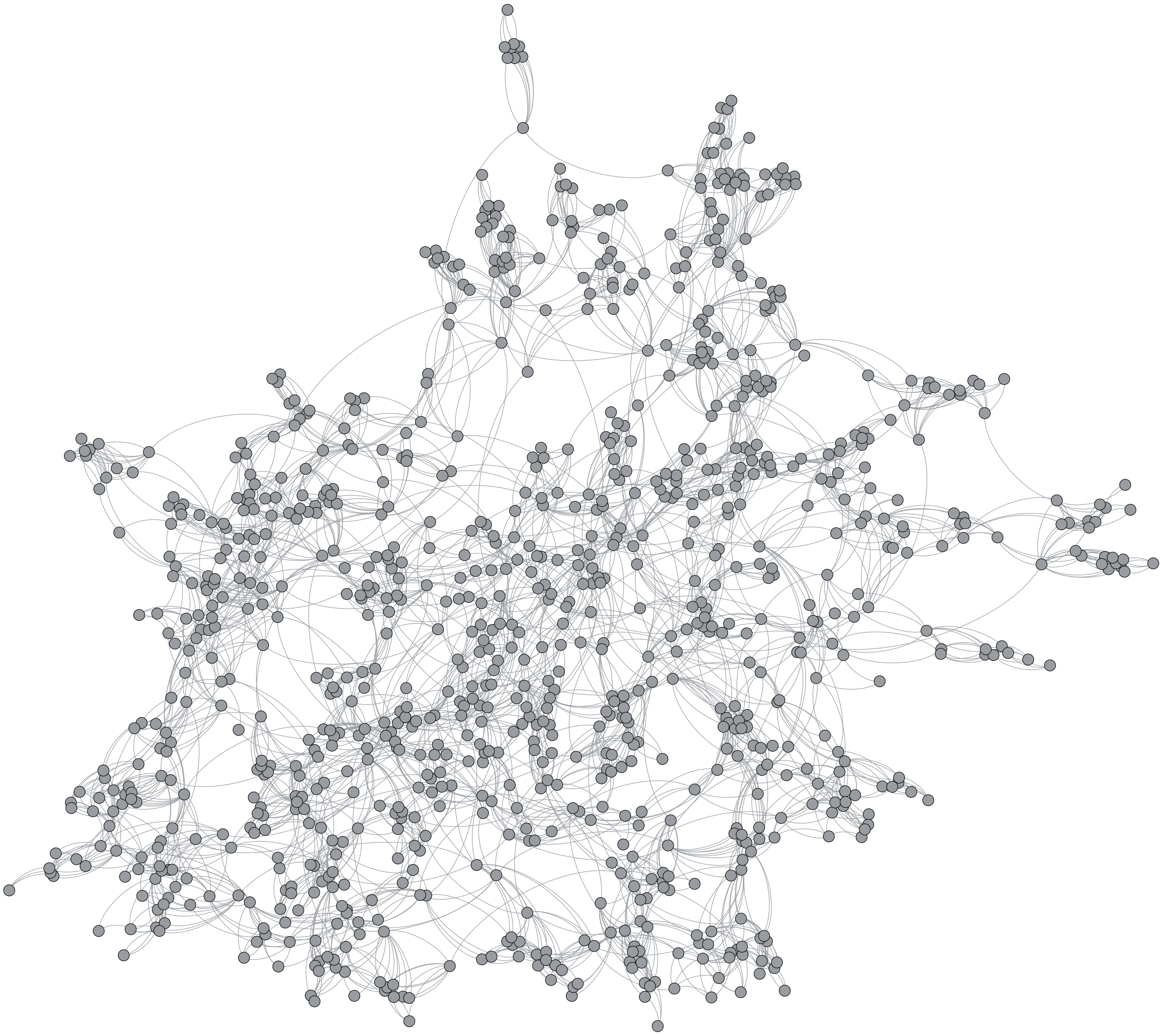

FIG. 4: (Color online.) **Topological structure of the network rewired from the ER network of Fig. 1 by the Kim's rewiring approach**. The rewired network has the clustering coefficient $C \approx 0.7$.

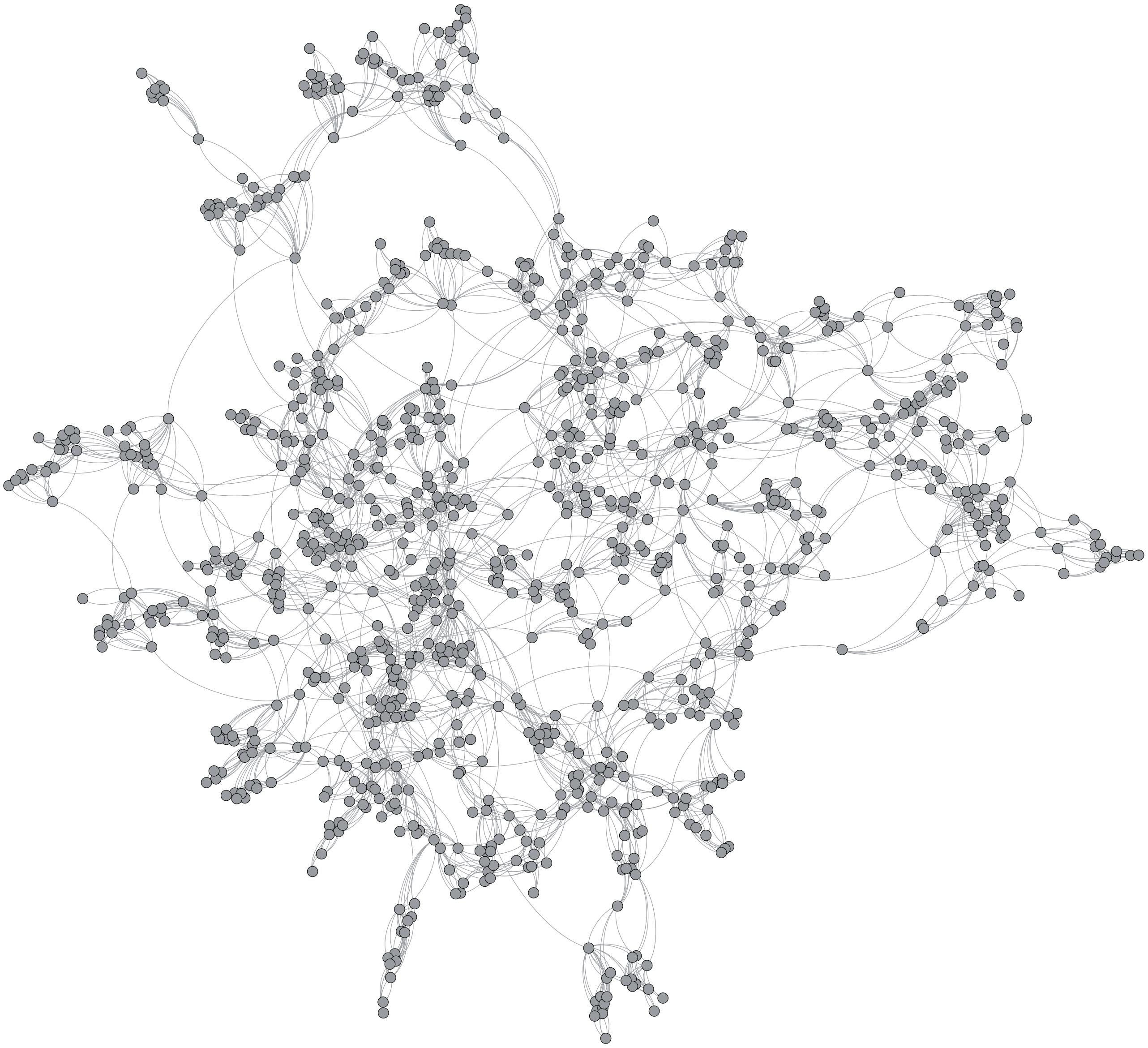

FIG. 5: (Color online.) **Topological structure of the network rewired from the ER network of Fig. 1 by the Kim's rewiring approach**. The rewired network has the clustering coefficient $C \approx 0.72$.

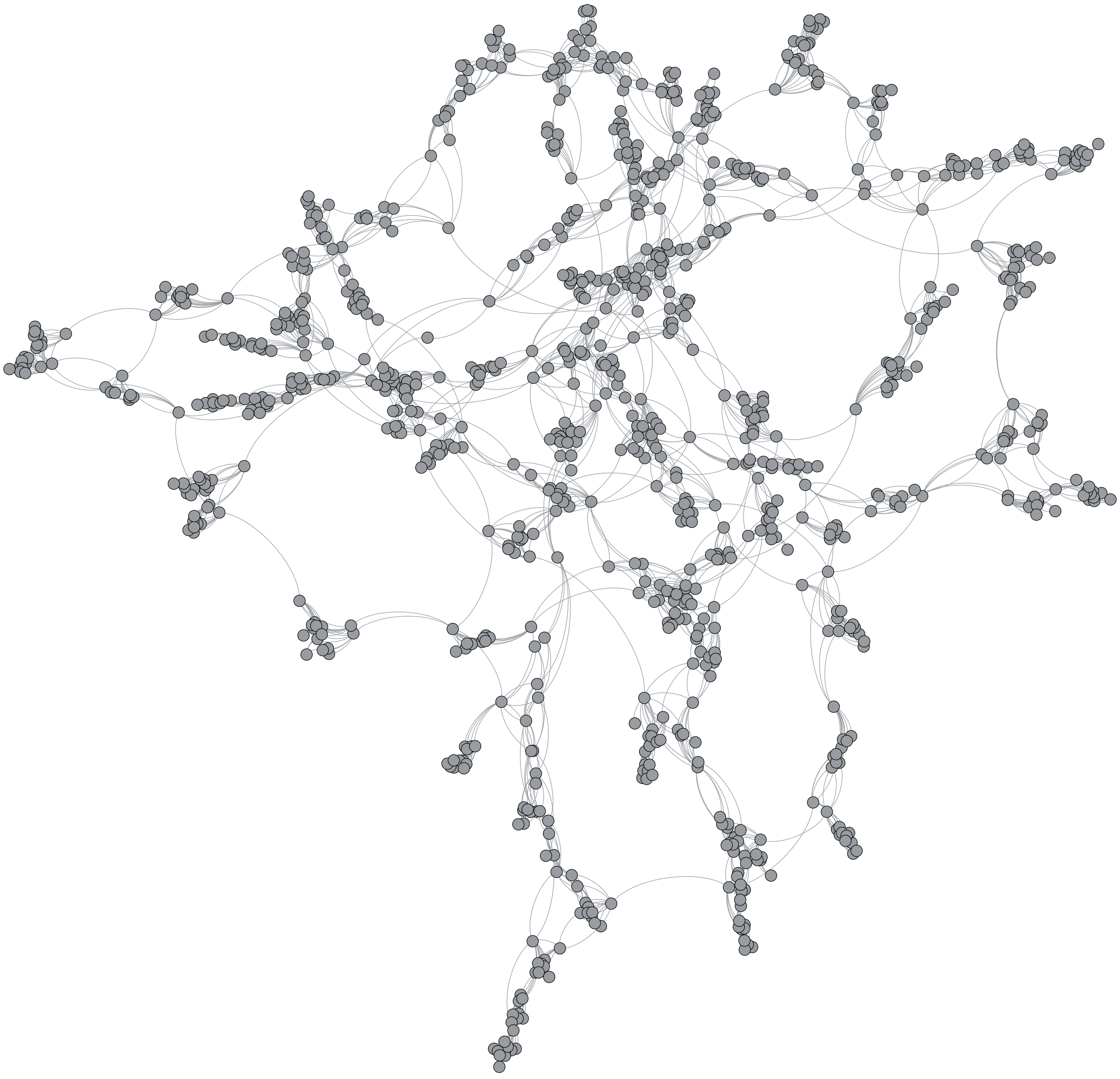

FIG. 6: (Color online.) **Topological structure of the network rewired from the ER network of Fig. 1 by the Kim's rewiring approach**. The rewired network has the clustering coefficient $C \approx 0.75$.

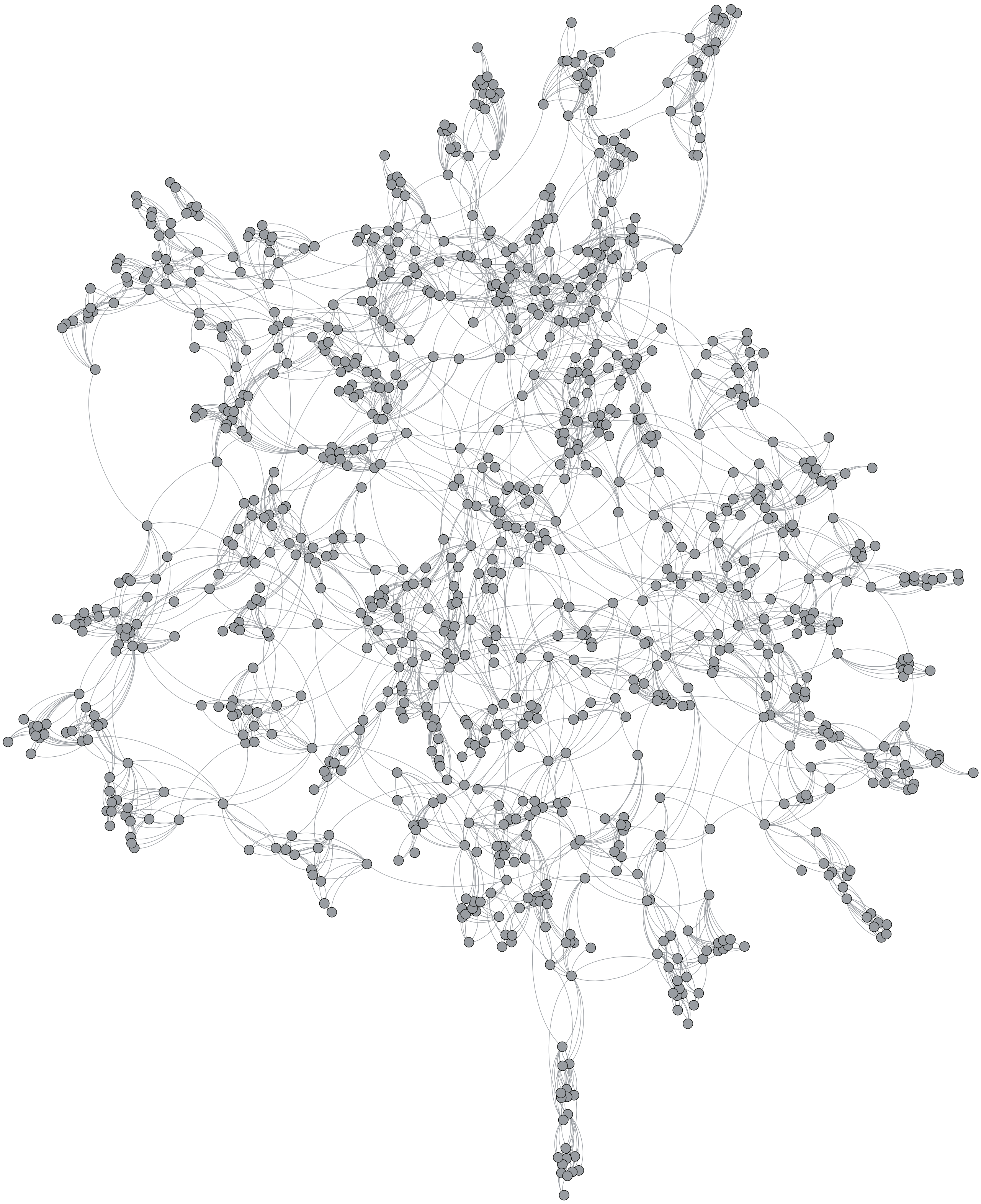

FIG. 7: (Color online.) **Topological structure of the network rewired** 40 **times from the network of Fig. 6 with clustering coefficient** $C = 0.75$ **by the random rewiring approach**. The network has size $N = 1000$, average degree $\langle k \rangle = 8$ and clustering coefficient $C \approx 0.72$.

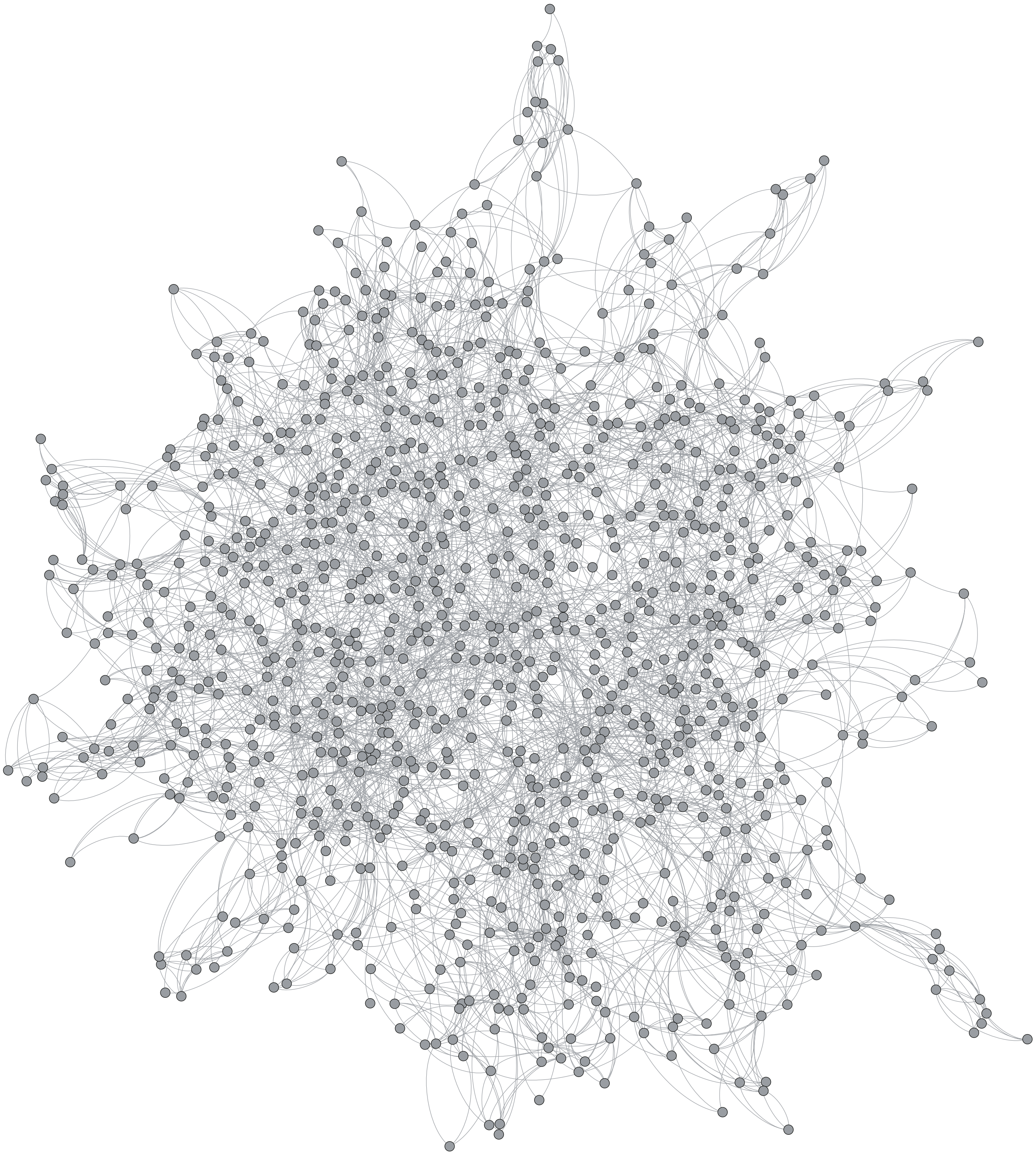

FIG. 8: (Color online.) **Topological structure of the network rewired** 300 **times from the network of Fig. 6 with clustering coefficient** $C = 0.75$ **by the random rewiring approach**. The network has size $N = 1000$, average degree $\langle k \rangle = 8$ and clustering coefficient $C \approx 0.5$.

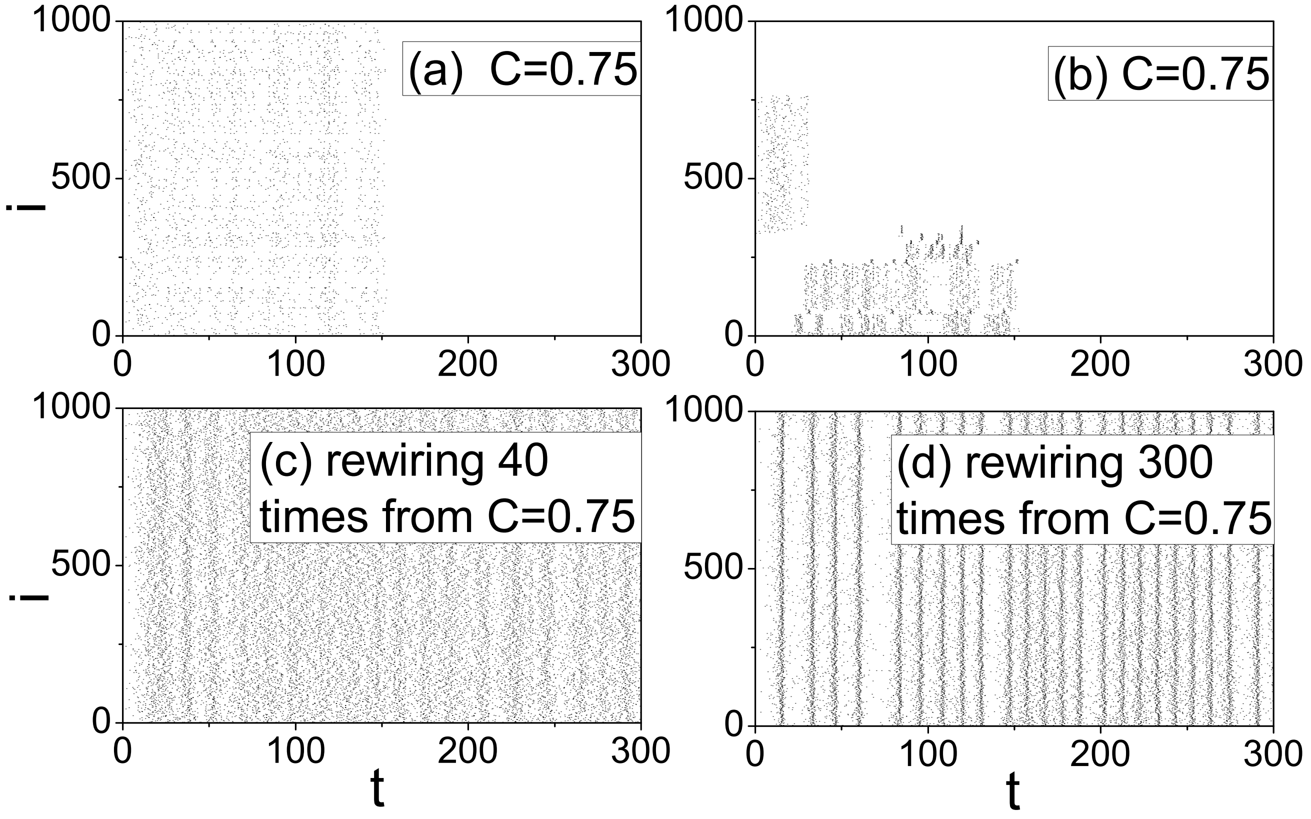

FIG. 9: (Color online.) **Firing spreading for different network topologies**. Evolution of an initial firing in the network with size $N = 1000$ and average degree $\langle k \rangle = 8$. A point is put in the figure when there is a firing at node-i and leave it empty when there is no firing. (a) represents the case of $C \approx 0.75$. (b) is the reordering of (a) by the total number of firings at each node in the evolution period, with descending order. (c) and (d) represent the cases of randomly rewiring the network of (a) for 40 and 300 times, respectively, where the rewired network has $C \approx 0.72$ and 0.5, respectively.

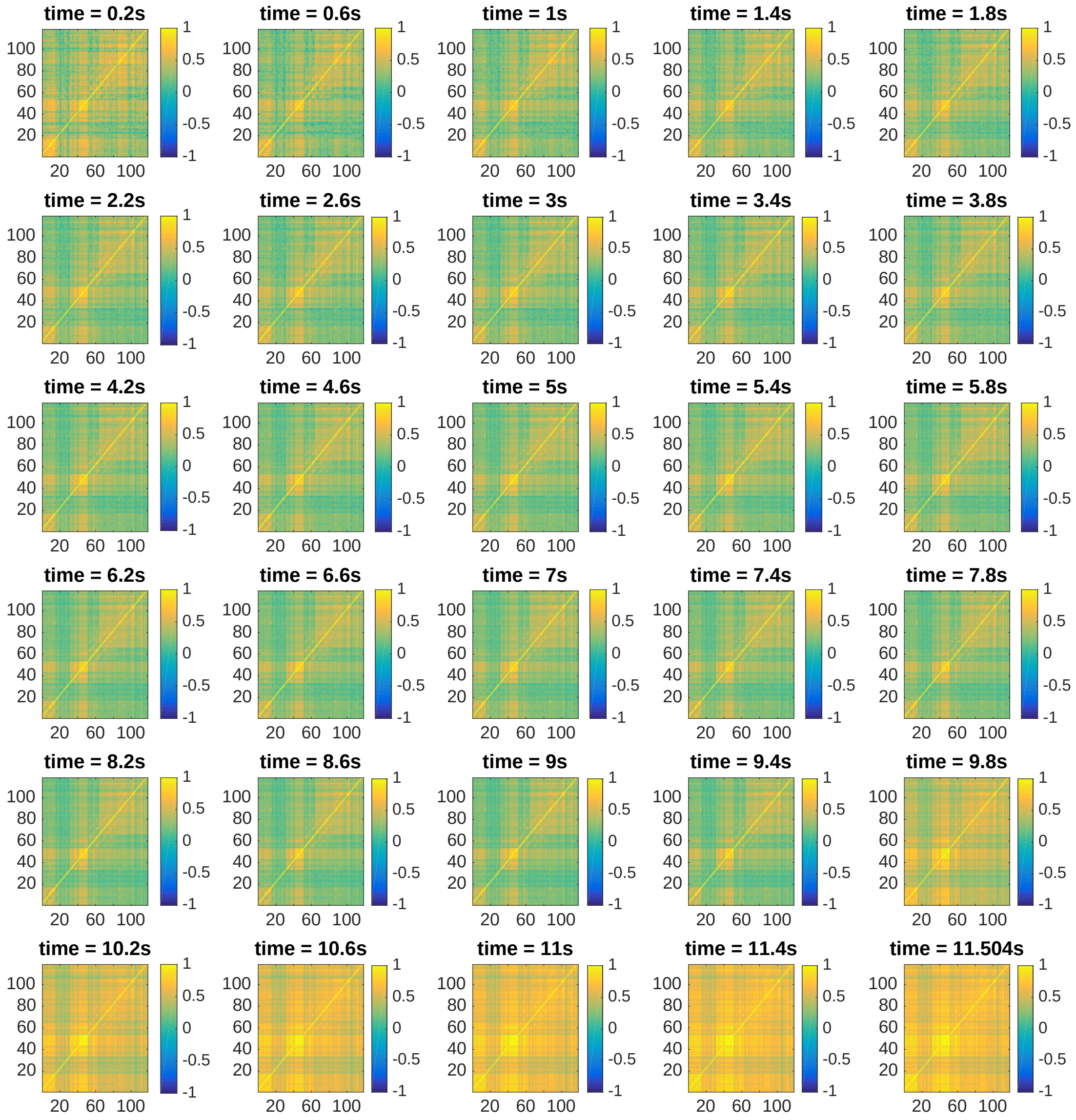

FIG. 10: (Color online). Evolution of phase correlation matrix from experimental data where the time interval is taken as $0.4s$. The elements of R_{ij} is defined in the *Methods* of main text. The time series to obtain R_{ij} are shown in Fig. 4 of main text.
